# Risk factors and predictive performance for first healthcare encounter indicating homelessness using administrative data among Calgary residents diagnosed with addiction or mental health conditions

**DOI:** 10.1371/journal.pdig.0001064

**Published:** 2025-10-31

**Authors:** Faezehsadat Shahidi, M. Ethan MacDonald, Dallas Seitz, Rebecca Barry, Geoffrey Messier

**Affiliations:** 1 Electrical and Software Engineering, Schulich School of Engineering, University of Calgary, Calgary, Alberta, Canada; 2 Biomedical Engineering, Schulich School of Engineering, University of Calgary, Calgary, Alberta, Canada; 3 Hotchkiss Brain Institute, University of Calgary, Calgary, Alberta, Canada; 4 Psychiatry, Cumming School of Medicine, University of Calgary, Calgary, Alberta, Canada; University of Alberta, CANADA

## Abstract

Individuals diagnosed with addiction or mental health (AMH) conditions are more likely to experience potentially adverse outcomes of homelessness. Despite their link to later outcomes, research on initial episodes of AMH outcomes is limited. This study aims to use administrative data to identify the factors associated with the first healthcare encounters with indicators of homelessness (FHE-H) for individuals diagnosed with AMH. We assessed logistic regression and compared its performance with machine learning models, including random forests and extreme gradient boosting (XGBoost). We conducted a retrospective cohort study linking several administrative datasets for 232,253 individuals with Alberta health insurance in Calgary, Canada, who were aged between 18 and 65 and diagnosed with AMH between April 1, 2013, and March 31, 2018. We assessed outcomes in two years following cohort entry. Individuals with episodes of FHE-H (2,606 individuals) before the index date were excluded. Multivariable logistic regression models were used to identify factors associated with outcomes by estimating adjusted odds ratios (AORs) with 95% confidence intervals. Among 229,647 individuals diagnosed with AMH, 1,886 (0.82%) experienced FHE-H during the follow-up period. Mental health emergency visits (AORs=5.28 [95% CI: 4.41, 6.33]), substance misuse (AORs=3.87 [95% CI: 3.28, 4.56], substance use disorders (AORs=2.03 [95% CI: 1.64, 2.50]), and male sex (AORs=1.28 [95% CI: 1.14, 1.44]) were associated with FHE-H. XGBoost performance improved over logistic regression, with increases in area under the curve (AUC) by 1% and precision by 2%. Overall, several AMH features were associated with FHE-H, and machine learning models outperformed logistic regression, although to a small degree.

## Author summary

Individuals diagnosed with addiction or mental health (AMH) conditions face a higher risk of experiencing homelessness, yet little is known about the early indicators that precede this outcome. In this study, we analyzed administrative healthcare data from over 230,000 adults in Calgary, Canada, to identify factors linked to a first healthcare encounter indicating homelessness. Using both statistical and machine learning models, we found that emergency mental health visits, substance misuse, and substance use disorders were the strongest predictors of future homelessness risk. Although machine learning only modestly improved prediction accuracy, it provided deeper insight into how multiple factors interact to shape vulnerability. By identifying individuals at high risk earlier, our findings highlight opportunities for preventive interventions and improved care coordination. This research demonstrates how large-scale administrative healthcare data can be used to better understand the factors associated with experiencing homelessness among people diagnosed with AMH conditions.

## Introduction

Individuals diagnosed with addiction or mental health (AMH) conditions [1] are at higher risk of negative outcomes, such as homelessness, a high-cost and high-risk issue in the developed world [2]. Although homelessness is relatively rare, its serious consequences make it important to identify characteristics that help predict this outcome [3]. Given the strong link between emergency shelter use and future homelessness [4], identifying factors associated with first-time healthcare encounters for homelessness (FHE-H) is essential for targeted prevention [5]. Individuals diagnosed with AMH conditions often face elevated rates of healthcare services. Healthcare administrative data contains information about healthcare encounters, including indicators of physical health, AMH conditions, and homelessness. Traditional logistic regression models may struggle to predict rare events effectively, and machine learning methods may offer improved performance for administrative data [6].

According to the World Health Organization, one in four people globally experiences mental health problems [7]. Based on Government of Canada reports, mental health conditions were experienced by 1 in 3 Canadians during their lifetime [8]. Various risk factors, including demographic characteristics such as male sex [9,10] and age [11], health service utilization [12,13], and physical and mental comorbidities [10,14–16], were found to be associated with homelessness in patients diagnosed with AMH conditions.

A person experiencing homelessness refers to someone who lacks stable, safe, and adequate housing [17]. Approximately 150 million people are experiencing homelessness globally at any given time [18]. Each year, approximately 235,000 Canadians experience homelessness [19], with 77% of them having lifetime mental health disorders [20].

The logistic regression model is the standard model used for exploring predictors of adverse outcomes due to its simplicity [21]. The logistic regression model has limitations when used for predicting outcomes, including difficulties in handling continuous variables, restrictions on the number of input variables, and challenges in managing highly correlated predictors [22]. Additionally, a comprehensive survey found that greater class imbalance in data significantly impacts model performance [23]. Balancing methods, such as random over-sampling of the training set, are shown to improve performance [24,25].

Two widely used machine learning models in previous studies for administrative data are random forests [26] and extreme gradient boosting (XGBoost) [27], which can overcome the limitations of the logistic regression model. The random forests model operates by aggregating decisions from multiple decision trees, each based on randomly selected variables in the dataset [28]. XGBoost, on the other hand, is based on a decision-tree ensemble algorithm that uses a gradient-boosting framework [29]. Nonlinear machine learning models generally exhibit superior predictive performance compared to logistic regression models [26], but some studies found that a linear model could outperform machine learning models while using administrative data [6].

We hypothesize that specific demographic, clinical, and healthcare utilization factors are associated with FHE-H among individuals with a history of AMH diagnoses. Additionally, we hypothesize that machine learning models, when trained with multinomial features, normalized data, and balanced classes, will demonstrate better predictive performance, measured by the area under the curve (AUC), sensitivity, and precision, compared to traditional logistic regression.

The main objective of this study is to identify factors associated with the FHE-H among individuals with a documented history of AMH diagnoses by linking several administrative healthcare datasets in Calgary, Canada. With a focus on a population diagnosed with AMH, we aim to assess whether machine learning models, such as random forests and XGBoost, offer meaningful improvements in predictive performance compared to logistic regression models in the context of AMH. Multinomial features, normalized data, and balanced classes are used to train the models. The AUC derived from the Receiver Operating Characteristic curve, along with sensitivity and precision, is used to assess performance.

## Methods

### Study design and data access

The data selected enabled a retrospective cohort descriptive study using secondary data. The manuscript followed the observational routinely collected data (RECORD) reporting guideline (S1 Appendix) [30]. The study protocol was reviewed and approved by the University of Calgary Ethics REB21-0070; Data Disclosure Agreements were developed with Alberta Health Services (AHS) and Alberta Health.

### Setting and years of data

To gain insight into the local epidemiology and resource utilization implications of AMH diagnoses, a population-based study was conducted using several healthcare administrative datasets collected between April 1, 2013, and March 31, 2020. The study population included individuals between the ages of 18 and 65 diagnosed with AMH as of April 1, 2018. The dataset included all Calgary residents who were alive and covered by Alberta Health Care Insurance, which provides access to medically necessary services, including physician visits, hospitalizations, and emergency room care. All residents of Alberta, including those with both lower and higher incomes, are covered under Alberta Health Care Insurance. To determine the optimal size of the observation and prediction windows, as well as the associated factors, some studies rely on the model performance by using various window lengths [31,32]. Similar long-term frameworks have been successfully applied in addiction and mental health research, such as the five-year post-treatment design by Hubbard et al. [33]. The subsequent two-year follow-up aligns with critical risk periods commonly used in psychiatric and addiction relapse research and balances outcome incidence with model validity [34].

### Data sources

We used several administrative health databases, including the Discharge Abstract Database (DAD), National Ambulatory Care Reporting System (NACRS), Practitioner Claims (claims), Pharmaceutical Information Network (PIN), Alberta Vital Statistics (AVS), and Registry database during the study period.

The AMH conditions, chronic conditions, and comorbid diagnoses [35] were partially derived from the DAD. Previous hospitalizations, previous hospitalizations for AMH conditions, previous hospitalizations for non-AMH conditions, and healthcare encounters with indications of homelessness were acquired from the DAD database [36]. Outpatient data, including previous ambulatory visits, elective and nonelective mental health-related emergency department (ED) visits (elective visits can be planned or postponed if needed, while nonelective were performed immediately because of an urgent or life-threatening medical condition), healthcare encounters with the indications of homelessness were accessed from NACRS [37]. AMH diagnoses, physical diagnoses, and previous physician visits (including family general practitioners, Internal medicine physicians, neurologists, psychiatrists, and others) were accessed from claims. Pharmaceutical and drug dispensation records for cognitive mental health diagnoses were collected from the PIN. The all-cause mortality data were gained from the AVS database [38]. Insurance status in Alberta was obtained from the Registry database.

### Data linkage

We used several linked administrative health databases and an electronic health record. The data used in this study were extracted from the Alberta Health Services Enterprise Data Warehouse, with support provided by the Alberta Strategy for Patient-Oriented Research Support Unit (AbSPORU). The data were linked using scrambled versions of the personal health number (PHN). Patient identifiers, names, dates of birth, and postal codes were all removed by the AHS. All the codes used in this study were included in the supplementary S2 Appendix. The flowchart diagram depicting the cohort creation, data cleaning, and analyses was included in the S3 Appendix.

### Cohort creation

The dataset consisted of a total of 232,253 unique individuals diagnosed with AMH during the study period in Calgary, Alberta. AMH conditions were defined for cohort entry based on DAD and Claims within two years (but at least 30 days apart) for any AMH diagnoses. We created the cohort with a 5-year fixed observation window and a 2-year fixed follow-up (outcome) window. The individuals with healthcare encounters indicating homelessness before the index date were excluded. So, individuals who experienced FHE-H outcome before March 31, 2018 (or during the observation window) were excluded from the analysis, like patients 2 and 4 in the S4 Appendix.

The observation and prediction window length were defined based on previous studies [33,34]. We included all Calgary residents diagnosed with AMH, covered by provincial health care insurance, who were alive between April 1, 2013, and March 31, 2018. We then followed them from March 31, 2018, to March 31, 2020, for the determination of the outcome. This cohort was used to identify factors associated with the FHE-H.

### Variables of interest and definitions

The International Statistical Classification of Diseases and Related Health Problems (ICD) [39], specifically ICD-9 codes, were applied to the claims data, and ICD-10 codes for the DAD and the NACRS were used as diagnostic coding systems to classify and categorize medical conditions, including AMH diagnoses, health service utilization, and comorbidities. Refer to the S2 Appendix for the comprehensive ICD code list.

#### Primary outcomes.

The primary outcome was the FHE-H. Homelessness was defined as the first healthcare record containing ICD codes indicating homelessness, ICD-9 codes (V600, V601) for claims data, and ICD-10 codes (Z590, Z591) for the DAD and NACRS were identified and extracted for each participant. Refer to the S5 Appendix for more details.

#### Potential predictors.

Predictors were selected a priori as clinically meaningful factors within the timeframe of April 1, 2013, to March 31, 2018. These predictors included demographic characteristics (such as age and sex), mental and physical comorbidities (classified according to the Elixhauser index [35]), and AMH diagnoses, including substance use disorders, anxiety disorders, mood disorders, psychotic disorders, other psychiatric disorders, cognitive disorders, and deliberate self-harm. Health service utilizations, including physician visits, previous hospital, and ED visits (with the concern of mental or non-mental health problems), were included. Healthcare interactions were included regardless of the specific diagnosis recorded at the time of the visit, in order to capture overall patterns of healthcare utilization rather than only those directly related to psychotic disorders.

We used the Elixhauser Index to quantify overall comorbidity burden (refer to S6 Appendix for more details), a method shown to predict adverse outcomes among homeless patients and general inpatient populations with strong discrimination [40]. Psychiatric conditions, such as psychotic, mood, anxiety, and substance use disorders, were included due to their high prevalence and predictive value for homelessness in administrative data (e.g., schizophrenia present in 13.3% of homeless inpatients) [40–43]. We included counts of ED, hospital, and physician visits, aligning with evidence that combined clinical and utilization predictors significantly improve homelessness prediction models [41,44]. Age and sex were found to be associated with a high risk of homelessness [41,42].

### Statistical analysis

Descriptive statistics were employed to summarize the characteristics of the created cohort using the dichotomous features. Both mean (standard deviation [SD]) and median (interquartile range [IQR]) were reported for the description of normally and non-normally distributed data [45]. We reported frequencies and percentages for categorical variables. Means with standard deviations (SD) were reported for normally distributed continuous variables, while medians with interquartile ranges (IQRs) were reported for skewed continuous variables.

We employed the univariate logistic regression model to estimate odds ratios (ORs) with 95% confidence intervals (CI) to identify predictive factors for FHE-H outcome with a 5-year observation window and a 2-year follow-up window [46]. We then applied multivariable logistic regression, using all predictors listed in S6 Appendix, to examine the joint association of risk factors with FHE-H outcomes. The forward selection method was used to add the variables to the predictive models [47]. To validate the predictive models, we randomly split the data based on the individuals into a training set (90% of the sample data) and a test set (10% of the sample).

### Predictive machine learning model development

Three different logistic regression models were trained to predict the first occurrence of homelessness. Model 1 used a 0.5 threshold without any additional techniques, while Model 2 used the Youden index to calculate the optimal cut-off point for the logistic regression model. The critical weakness of predictive analytics was found to be the cut-off point when evaluating logistic regression models [48]. The Youden index approach [49] was applied to calculate the optimal cut-off point by measuring the difference between the true positive rate and the false positive rate across all potential values [50,51]. Both Model 1 and Model 2 were trained using dichotomous features (0, +1) in the observation window. Model 3 was compared with machine learning models in terms of performance.

During preprocessing for Model 3 and the machine learning models, multinomial features were utilized alongside normalization techniques, such as power transformation, and a balancing method like the random over-sampling technique for the training set [24,25]. The test set remained unaltered for the final performance evaluation across all experiments.

The machine learning models, including random forests and XGBoost, were evaluated for predicting the FHE-H [26,29,52] to be compared with Model 3. We submitted several jobs in parallel as array jobs [53]. Each job had a unique input to do hyperparameter optimization. The best results received by these hyperparameters were illustrated in the supplementary S7 Appendix.

The machine learning models and logistic regression (Model 3) were examined using multinomial features, normalized data, and balanced classes. The previous studies found that the model performance was affected by the rate of class imbalances [23]. The random over-sampling method to balance classes and power transformation to normalize data improved the performance while using Model 3 (logistic regression), random forests, and XGBoost models [23,54–56]. Data analysis and model development were performed using the Python programming language (version 3.9).

## Results

Of 232,253 individuals, 232,023 were diagnosed with AMH based on DAD and Claims. Additionally, 31,248 individuals diagnosed with AMH conditions (cognitive mental diagnoses) were included using PIN data. Individuals with healthcare encounters indicating homelessness outcome before the index date were excluded, resulting in the removal of 2,606 individuals (1.12%) with the FHE-H outcome. The final dataset consisted of 229,647 individuals (S8 Appendix). Out of 229,647 individuals, 1,886 (0.82%) experienced their FHE-H during the prediction window. Of those, 1,552 individuals were identified from NACRS, and 334 were extracted from DAD using healthcare ICD codes.

### Predictors of outcome among individuals diagnosed with AMH

In a cohort of 229,647 individuals, 1,886 (0.82%) experienced their FHE-H. Among those, 1,206 (63.9%) were male, with a median age of 37 years (interquartile range: 21 years). A total of 1,626 (86.2%) had a diagnosis of substance use disorder, and 1,456 (77.2%) were diagnosed with an anxiety disorder. Notably, 1,871 (99.2%) had general practitioner visits and 1,868 (99.0%) had no mental health-related emergency room visits. The most prevalent comorbidities were depression (1,617 (85.7%) and a history of substance misuse (1,462 (77.5%)) (Table 1).

**Table 1:** Characteristics of individuals in Calgary, Alberta, Canada who were diagnosed with AMH between April 1, 2013, and March 31, 2018, and the difference between those who experienced FHE-H during the two-year follow-up period and those who did not.

Characteristic		The FHE-H outcome		
		Yes=1,886(0.82)	No=227,761(99.2)	Total=229,647
Sex	Male	1,206 (63.9)	97,087 (42.6)	98,293 (42.8)
	Female	680 (36.1)	130,674 (57.4)	131,354 (57.2)
Age		[IQR:21]	[IQR:22]	[IQR:22]
		[median:37]	[median:42]	[median:42]
Age category	18-29	506 (26.8)	43,961 (19.3)	44,467 (19.4)
	30-39	545 (28.9)	54,757 (24.0)	55,302 (24.1)
	40-49	363 (19.2)	51,261 (22.5)	51,624 (22.5)
	50-59	350 (18.6)	51,047 (22.4)	51,397 (22.4)
	60+	122 (6.5)	26,735 (11.7)	26,857 (11.7)
AMH features	Substance use disorders	1,626 (86.2)	48,992 (21.5)	50,618 (22.0)
	Anxiety disorder	1,456 (77.2)	163,012(71.6)	164,468 (71.6)
	Mood disorder	1,381 (73.2)	106,547 (46.8)	107,928 (47.0)
	Other psychiatric disorders	868 (46.0)	70,071 (30.8)	70,939 (30.9)
	Psychotic disorder	577 (30.6)	10,374 (4.6)	10,951 (4.8)
	Cognitive disorders	426 (22.6)	30,295 (13.3)	30,721 (13.4)
	Deliberate self-harm	112 (5.9)	1,724 (0.8)	1,836 (0.8)
Health service utilization	General practitioner	1,871 (99.2)	226,167 (99.3)	228,038 (99.3)
	ED non-MH	1,868 (99.0)	203,704 (89.4)	205,572 (89.5)
	Other physicians	1,866 (98.9)	221,176 (97.1)	223,042 (97.1)
	ED MH	1,664 (88.2)	41,199 (18.1)	42,863 (18.7)
	Psychiatrist	1,501 (79.6)	70,418 (30.9)	71,919 (31.3)
	Internal medicine	1,367 (72.5)	144,117 (63.3)	145,484 (63.4)
	ED (general)	1,131 (60.0)	50,522 (22.2)	51,653 (22.5)
	MH hospitalized	940 (49.8)	14,874 (6.5)	15,814 (6.9)
	Non-MH hospitalized	850 (45.1)	9,882 (4.3)	10,732 (4.7)
	Neurologist	328 (17.4)	30,489 (13.4)	30,817 (13.4)
Elixhauser comorbidities	Depression	1,617 (85.7)	147,720 (64.9)	149,337 (65.0)
	Substance misuse	1,462 (77.5)	21,437 (9.4)	22,899 (10.0)
	Tobacco use	1,181 (62.6)	48,321 (21.2)	49,502 (21.6)
	Alcohol use	1,041 (55.2)	11,483 (5.0)	12,524 (5.5)
	Psychotic disorders	923 (48.9)	18,286 (8.0)	19,209 (8.4)

Abbreviations: FHE-H, first healthcare encounter with an indication of homelessness; AMH, addiction or mental health; ED, emergency department; MH, mental health; IQR, interquartile range.

In our multivariable analysis of individuals within two years of their FHE-H experience, several variables were associated with the FHE-H (Table 2). In terms of demographic characteristics, males had adjusted odds ratios (AORs) of 1.28 [95% CI: 1.14, 1.44] (p < 0.05) compared to females. Individuals aged 60 and above had an odds ratio of 0.78 [95% CI: 0.61, 0.99] (p < 0.05), indicating a lower risk compared to the reference group (30-39 years old). Among the AMH conditions, substance use disorders and psychotic disorders exhibited the highest AORs, 2.03 [95% CI: 1.64, 2.50] and 1.22 [95% CI: 1.04, 1.43], respectively, with p-values < 0.05, indicating strong associations with the FHE-H outcome. Regarding health service utilization, ED visits for mental health (AORs = 5.28 [95% CI: 4.41, 6.33], p < 0.05) and non-mental health (AORs = 4.21 [95% CI: 2.54, 6.96], p < 0.05) reasons, along with general visits (AORs = 1.21 [95% CI: 1.06, 1.37], p < 0.05), were associated with higher and stronger odds of the FHE-H. Non-mental health hospitalization had the AORs of 1.91[95% CI: 1.67, 2.18] (p < 0.05). Among all comorbidities, the AORs of substance misuse and alcohol use were 3.87 [95% CI: 3.28, 4.56] (p < 0.05) and 1.70 [95% CI: 1.50, 1.93] (p < 0.05), respectively, indicating significant associations with the FHE-H outcome. Human immunodeficiency virus (HIV) or acquired immunodeficiency syndrome (AIDS) and psychotic disorders were strongly associated with FHE-H outcome, with p-values < 0.05 and AORs of 2.00 [95% CI: 1.27, 3.14] and 1.66 [95% CI: 1.43, 1.94], respectively.

**Table 2:** Associations between clinical risk factors and the FHE-H during a period of seven years, April 1, 2013, to March 31, 2020, diagnosed with AMH. Individuals were observed for five years and subsequently followed up for two years.

Characteristic		Univariate		Multivariable	
		ORs(95%CI)	*P*–*value*	AORs(95%CI)	*P*–*value*
Sex	Male	2.39(2.17,2.62)	<0.05	1.28(1.14,1.44)	<0.05
	Female	1[*Reference*]	–	1[*Reference*]	–
Age category	60+	0.52(0.43,0.63)	<0.05	0.78(0.61,0.99)	<0.05
	50-59	0.79(0.7,0.89)	<0.05	0.94(0.79,1.11)	0.44
	40-49	0.82(0.73,0.92)	<0.05	0.96(0.82,1.12)	0.60
	30-39	1[*Reference*]	–	1[*Reference*]	–
	18-29	1.53(1.38,1.7)	<0.05	0.87(0.76,1.01)	0.06
AMH feature	Substance use disorder	22.82(20.01,26.02)	<0.05	2.03(1.64,2.50)	<0.05
	Psychotic disorder	9.24(8.36,10.21)	<0.05	1.22(1.04,1.43)	<0.05
	Mood disorder	3.11(2.81,3.45)	<0.05	1.12(0.96,1.31)	0.16
	Other psychiatric disorders	1.92(1.75,2.10)	<0.05	1.01(0.89,1.13)	0.93
	Anxiety disorder	1.34(1.21,1.50)	<0.05	0.88(0.76,1.01)	0.08
	Cognitive disorders	1.90(1.71,2.12)	<0.05	0.84(0.74,0.96)	<0.05
	Deliberate self-harm	8.28(6.80,10.08)	<0.05	0.62(0.49,0.79)	<0.05
Health service utilization	ED MH	33.94(29.49,39.06)	<0.05	5.28(4.41,6.33)	<0.05
	ED non-MH	12.26(7.70,19.50)	<0.05	4.21(2.54,6.96)	<0.05
	Non-MH hospitalized	18.09(16.48,19.85)	<0.05	1.91(1.67,2.18)	<0.05
	General practitioner	0.88(0.53,1.46)	<0.05	1.32(0.49,3.58)	0.59
	ED (general)	5.26(4.79,5.77)	<0.05	1.21(1.06,1.37)	<0.05
	Psychiatrist	8.71(7.79,9.75)	<0.05	1.15(0.97,1.35)	0.11
	MH hospitalized	14.22(12.97,15.59)	<0.05	1.11(0.97,1.26)	0.13
	Other physicians	2.78(1.79,4.32)	<0.05	1.09(0.47,2.54)	0.84
	Internal medicine	1.53(1.38,1.69)	<0.05	1.05(0.92,1.20)	0.45
	Neurologist	1.36(1.21,1.54)	<0.05	0.70(0.59,0.82)	<0.05
Elixhauser comorbidities	Substance misuse	33.19(29.76,37.01)	<0.05	3.87(3.28,4.56)	<0.05
	HIV/AIDS	6.93(4.78,10.04)	<0.05	2.00(1.27,3.14)	<0.05
	Alcohol use	23.20(21.15,25.46)	<0.05	1.70(1.50,1.93)	<0.05
	Psychotic disorder	10.98(10.02,12.03)	<0.05	1.66(1.43,1.94)	<0.05
	Fall	3.83(3.50,4.20)	<0.05	1.52(1.36,1.69)	<0.05

Abbreviations: FHE-H, first healthcare encounter with an indication of homelessness; AMH, addiction or mental health; ED, emergency department; MH, mental health; HIV/AIDS, human immunodeficiency virus or acquired immunodeficiency syndrome.

### Model performance

After implementing the Youden index and comparing Model 1 and Model 2, we observed an increase in sensitivity, precision, and AUC scores (Table 3). Model 2 (logistic regression model trained with dichotomous features, optimized by the Youden index approach, without using normalization) was used for univariate and multivariable analyses. As Model 2 gained better performance, it was used for univariate and multivariate analyses.

**Table 3:** The performance of all the predictive models for the FHE-H outcome.

	Model	Type	Optimization	Normalization	Precision	Sensitivity	AUC
Model 1	LR	DF	–	–	1%	33%	50%
Model 2	LR	DF	*J*	–	8%	83%	88%
Model 3	LR	MF	ROTE	PT	7%	87%	88%
Model 4	RF	MF	ROTE	PT	10%	83%	88%
Model 5	XGBoost	MF	ROTE	PT	9%	87%	89%

Abbreviations: FHE-H, first-time healthcare encounter indicating homelessness; LR, logistic regression; PT, power transformation for normalizing the data; ROTE, random over-sampling technique; *J*, Youden index; RF, random forest; XGBoost, extreme gradient boosting; AUC, the area under the curve; DF: dichotomous features; MF: multinomial features.

The XGBoost [57] and the random forests [26] were compared with Model 3 (logistic regression) for the FHE-H outcome. Multinomial features, the power transformation technique [58], and the random over-sampling method were utilized in the preprocessing phase. Both the random forests and XGBoost models slightly outperformed the logistic regression model. This finding aligns with previous studies [6], which also reported improvements with machine learning models. Our results showed that XGBoost performed similarly to logistic regression in terms of sensitivity (87%) and demonstrated a 1% increase in AUC (89%) compared to both logistic regression and random forests models. The XGBoost also showed a 2% improvement in precision over logistic regression. The random forests achieved the highest precision overall, with a 3% increase compared to logistic regression.

## Discussion

Several features were found to be associated with FHE-H outcome among residents of Calgary, Alberta, who were identified as having an AMH using administrative healthcare data. Combining these variables achieved a superior estimation in this population than if they were considered individually. For individuals who experienced their FHE-H, we observed high rates of anxiety disorders, substance use disorders, psychotic disorders, and depression [44,59,60]. The ED visits were the most frequent form of service utilization.

Our findings regarding the FHE-H were aligned with previous research, which identified various risk factors, including demographic characteristics such as male sex [9,10] and age [11]; AMH features such as substance use disorders [14,61,62] and psychotic disorders [12,63]; health service utilization, including mental health ED visits [12] and non-mental health-related ED and hospital visits [13]; mental comorbidities such as substance misuse [14,15], alcohol use [15], psychotic disorders [16,60,64]; and physical comorbidities such as HIV/AIDS [10] and histories of falls [65], which were previously recognized as risk factors for homelessness in patients diagnosed with AMH.

A key finding of this study is the strong association between substance use disorders and FHE-H, highlighting the critical role of substance use in housing instability among individuals with AMH conditions [66]. This supports existing evidence and underscores the need to integrate housing support into addiction services. Additionally, both mental health and non-mental health ED visits were significant predictors of FHE-H, suggesting that frequent ED use may signal unmet needs [67] and offer opportunities for early intervention through either health-focused supports [66] or housing-based solutions [68].

We used the Youden index to assess the overall performance, which is distinct from calibration. We chose to utilize the cut-off point instead of calibration due to its simplicity and straightforward interpretation. In contrast, calibration entails intricate adjustments to model probabilities to align with observed outcomes, which can be more intricate and computationally intensive. Examining calibration within various risk strata would yield supplementary insights into the model’s performance.

We discovered the importance of a cut-off point (using the Youden index) for predictive modeling, consistent with previous studies [69]. We also found that using normalization (power transformation) [70] and balancing classes (random over-sampling) [71] improved the performance, which was aligned with previous studies [23,72]. According to these studies, the large degree of the imbalanced classes (in our dataset, the rate was highly imbalanced) lowered the sensitivity, and therefore, strategies to improve the model should be considered [23]. This study found that the machine learning models achieved the highest performance in terms of sensitivity, precision, and AUC, aligned with prior research [73].

There are several limitations to our study. First, the accuracy of our methods depends on the completeness of administrative healthcare records. Administrative datasets may contain missing or inaccurate information due to errors in data entry or incomplete reporting. Homelessness that occurs outside the healthcare setting (particularly before an individual’s first recorded healthcare contact) is not captured. Additionally, underreporting of diagnoses may lead to underestimation, as missing or inaccurate data can result in values lower than the true ones. Relying solely on ICD codes likely underestimates the true prevalence of individuals diagnosed with AMH experiencing initial homelessness. Second, the administrative datasets may be subject to selection bias, as they include only those who have interacted with the system. For example, marginalized groups, such as individuals without documented status, may be underrepresented, introducing bias and limiting the accuracy of findings for these populations. Third, this study does not address the complex impacts of stigmatization or employment disruption. Another limitation is the inability to establish causality, as we were not able to control for all confounders due to the nature of the data, and factors such as socioeconomic status, geographic location, race, or country of origin were not available for modeling. As a result, the observed associations may reflect the influence of unmeasured factors rather than direct causal relationships. Fourth, the cohort was restricted to individuals diagnosed with AMH. While this enhances the relevance for targeted interventions, it limits generalizability and prevents assessment of AMH as a differentiating predictor, as it excludes comparisons with individuals not diagnosed with AMH. The Calgary-based sample also limits generalizability and may not extend to regions with different healthcare systems, service structures, or population demographics. Fifth, the preprocessing methods and linking the large administrative datasets posed significant challenges due to the volume and complexity of the datasets. Balancing overfitting and underfitting was also difficult, as the performance of machine learning algorithms depends heavily on appropriate hyperparameter selection relative to the number of observations and features [74]. Sixth, predictive models may perpetuate or amplify existing biases in the training data, leading to potentially unfair or discriminatory outcomes [75]. Finally, while all models demonstrated reasonable sensitivity, precision was low, indicating a substantial number of false positives. This may limit the practical utility of the predictions, as false positives, despite the use of advanced machine learning models, could result in unnecessary interventions and wasted resources.

This study has several strengths. First, it utilizes several administrative datasets from over 230,000 individuals diagnosed with AMH conditions in Calgary, Canada, providing a large sample size capable of detecting small differences between groups. By including all Calgary residents who accessed healthcare in Alberta, the study minimizes selection bias compared to surveys [76]. It focuses on the FHE-H outcome and uses all recorded diagnostic codes, reducing the likelihood of misclassification compared to survey data [76]. Although the definition for homelessness has low precision, it exhibits high sensitivity and AUC, likely capturing individuals genuinely experiencing these conditions. This study addresses a gap in understanding the relationship between AMH and the FHE-H. Understanding the factors associated with the initial outcome is important for preventing subsequent episodes of homelessness. Lastly, the study compares machine learning and logistic regression models in predicting FHE-H outcomes, demonstrating that machine learning models perform only marginally better than logistic regression models.

## Conclusions

We found that demographic characteristics, AMH features, comorbid mental and physical conditions, and healthcare service utilization were all associated with the FHE-H outcome. Using multinomial features, normalization, balancing classes, and the machine learning models provided slightly better or comparable predictive performance relative to the logistic regression model. Future research will focus on advanced preprocessing techniques and deep learning models to further enhance predictive performance.

## Supporting information

S1 Appendix The RECORD checklist of items that were reported in this study. (PDF)

S2 Appendix Data sources and the codes in details. (PDF)

S3 Appendix The diagram flowchart of the study as described in the methodology. (PDF)

S4 Appendix The framework for observation and prediction windows with a fixed index date. (PDF)

S5 Appendix The outcomes and definitions. (PDF)

S6 Appendix Predictors that were used in multivariable analysis. (PDF)

S7 Appendix The hyperparameters for each machine learning model. (PDF)

S8 Appendix Summary of individuals with AMH and cohort exclusions. (PDF)
