# Supplementary material for: Risk factors and predictive performance for first healthcare encounter indicating homelessness using administrative data among Calgary residents diagnosed with addiction or mental health conditions
Source: PLOS Digit Health. 2025 Oct 31;4(10):e0001064. doi: 10.1371/journal.pdig.0001064 (PMC12578244; doi:10.1371/journal.pdig.0001064)
Supplement: S1 Appendix — (PDF) [file pdig.0001064.s001.pdf]

S1 Appendix: The RECORD checklist of items that were reported in this study.

	RECORD items	Location in manuscript
**Title and abstract**		
	RECORD 1.1: The type of data used should be specified in the title or abstract. When possible, the name of the databases used should be included.	The database was stated in the title and abstract.
	RECORD 1.2: If applicable, the geographic region and timeframe within which the study took place should be reported in the title or abstract.	The location was stated in the title and abstract.
	RECORD 1.3: If linkage between databases were conducted for the study, this should be clearly stated in the title or abstract.	The linkage was stated in the abstract.
**Methods**		
**Participants**	RECORD 6.1: The methods of study population selection (such as codes or algorithms used to identify subjects) should be listed in detail. If this is not possible, an explanation should be provided.	In the method of the study, the population selection was explained.
	RECORD 6.2: Any validation studies of the codes or algorithms used to select the population should be referenced. If validation was conducted for this study and not published elsewhere, detailed methods and results should be provided.	This is not applicable as our population was explained in RECORD 6.1.
	RECORD 6.3: If the study involved linkage of databases, consider use of a flow diagram or other graphical display to demonstrate the data linkage process, including the number of individuals with linked data at each stage.	In Appendix 3, we illustrated all the steps to show the steps we took to create the cohort.
**Variables**	RECORD 7.1: A complete list of codes and algorithms used to classify exposures, outcomes, confounders, and effect modifiers should be provided. If these cannot be reported, an explanation should be provided.	All the codes were reported in Appendix 2.
**Data access and cleaning methods**	RECORD 12.1: Authors should describe the extent to which the investigators had access to the database population used to create the study population.	All the data sources that we had access to were explained in the data source section of the method.
	RECORD 12.2: Authors should provide information on the data cleaning methods used in the study.	Data cleaning was explained in the methodology, and the steps clearly defined in Appendix 3.
**Linkage**	RECORD 12.3: State whether the study included person-level, institutional-level, or other data linkage across two or more databases. The methods of linkage and methods of linkage quality evaluation should be provided.	Person-level linkage was performed, and it is clearly stated in the linkage part in the methodology and illustrated in Appendix 3.
**Results**		
**Participants**	RECORD 13.1: Describe in detail the selection of the persons included in the study (i.e., study population selection) including filtering based on data quality, data availability and linkage. The selection of included persons can be described in the text and/or by means of the study flow diagram.	The selection of the persons all described in the results, and it was clearly illustrated in the flowchart in Appendix 3.
**Discussion**		
**Limitations**	RECORD 19.1: Discuss the implications of using data that were not created or collected to answer the specific research question(s). Include discussion of misclassification bias, unmeasured confounding, missing data, and changing eligibility over time, as they pertain to the study being reported.	The limitation of the study is clearly stated in the discussion.
**Other Information**		
	RECORD 22.1: Authors should provide information on how to access any supplemental information such as the study protocol, raw data, or programming code.	supplemental information is submitted to the journal. Data access and programming codes are available in the Data Availability section.
