# Supplementary material for: Risk factors and predictive performance for first healthcare encounter indicating homelessness using administrative data among Calgary residents diagnosed with addiction or mental health conditions
Source: PLOS Digit Health. 2025 Oct 31;4(10):e0001064. doi: 10.1371/journal.pdig.0001064 (PMC12578244; doi:10.1371/journal.pdig.0001064)
Supplement: S2 Appendix — (PDF) [file pdig.0001064.s002.pdf]

S2 Appendix: Data Sources and the Codes in Details.

Data Source	Codes	Details
1. DAD	ICD-10-CA for comorbidities	Type: DXCODE1 - DXCODE 25
	ICD-10-CA for substance use disorder	Any hospitalization: F10.X—F19.X, F55.X, F63.X
	ICD-10-CA for mood disorder	F30.X, F31.X, F32.X, F33.X, F34.X, F38.X, F39.X, F53.0
	ICD-10-CA for anxiety disorder	F40.X, F41.X, F42.X, F43.X, F48.8, F48.9
	ICD-10-CA for psychotic disorder	F06.0, F06.1, F06.2, F06.0-2, F20.X, F22.X, F23.X, F24.X, F25.X, F26.X, F27.X, F28.X, F29.X, F22-F29, F53.1
	ICD-10-CA for cognitive disorders	F00X, F01.X, F02.X, F03.X G30.X
	ICD-10-CA for Other psychiatric disorders	F06-F99
	ICD-10-CA for Deliberate self-harm	X60-X84, Y10-Y19, Y28 when DX10CODE1 was not equal to F06-F99
	ICD-10-CA for homelessness	Z590, Z591
	MPSERV for the number of hospitalizations to a designated psychiatric facility	64
	MPSERV number hospitalizations to non-psychiatric facility	Not equal to 64
2. Claims	ICD-9-CM for comorbidities	HLTH_DX_ICD9x_CODE_1, HLTH_DX_ICD9x_CODE_2 HLTH_DX_ICD9x_CODE_3
	ICD-9-CM for substance use disorder	2 or more physician claims in 2 years with diagnosis: 291, 292,303, 304, 305
	ICD-9-CM for mood disorder	2 physicians claim at least 30 days apart within 2 years with one or more of the diagnoses codes: 296, 311
	ICD-9-CM for anxiety disorder	2 physicians claim at least 30 days apart within 2 years with one or more of the diagnoses codes: 300, 308, 309
	ICD-9-CM for psychotic disorder	2 physicians claim at least 30 days apart within 2 years with one or more of the diagnoses codes: 295, 297, 298
	ICD-9-CM for cognitive disorders	3 physicians claim at least 30 days apart within 2 years with one or more of the diagnoses codes: 290, 331
	ICD-9-CM for Other psychiatric disorders	2 physicians claim at least 30 days apart within 2 years with one or more of the diagnoses codes: 290 – 319
	ICD-9-CM for homelessness	V600, V601
	PERS_CAPB_PRVD_SPEC_AD for Health Service Utilization	GP: family physician visits<br>NEUR: visits by neurologists<br>INMD: visits by general internal medicine<br>PSYC: visits by psychiatrists
3. PIN	DINS codes for cognitive disorders	Donepezil, Galantamine, Rivastigmine or Memantine
4- NACRS	ICD-10-CA for comorbidities (79)	Type: DXCODE1 - DXCODE 25
	ICD-10-CA for a fall-related injury	W01, W10, W11, W12, W13, W06-W08, W09.01-W09.05, W09.08, W09.09, W14-W17, W00, W02.00-W02.05, W02.08, W03, W04, W05.00-W05.04, W05.08, W05.09, W18, W19
	ICD-10-CA for homelessness	Z590, Z591
