# Supplementary material for: Risk factors and predictive performance for first healthcare encounter indicating homelessness using administrative data among Calgary residents diagnosed with addiction or mental health conditions
Source: PLOS Digit Health. 2025 Oct 31;4(10):e0001064. doi: 10.1371/journal.pdig.0001064 (PMC12578244; doi:10.1371/journal.pdig.0001064)
Supplement: S3 Appendix — (PDF) [file pdig.0001064.s003.pdf]

**S3 Appendix:** The diagram flowchart of the study as described in the methodology.

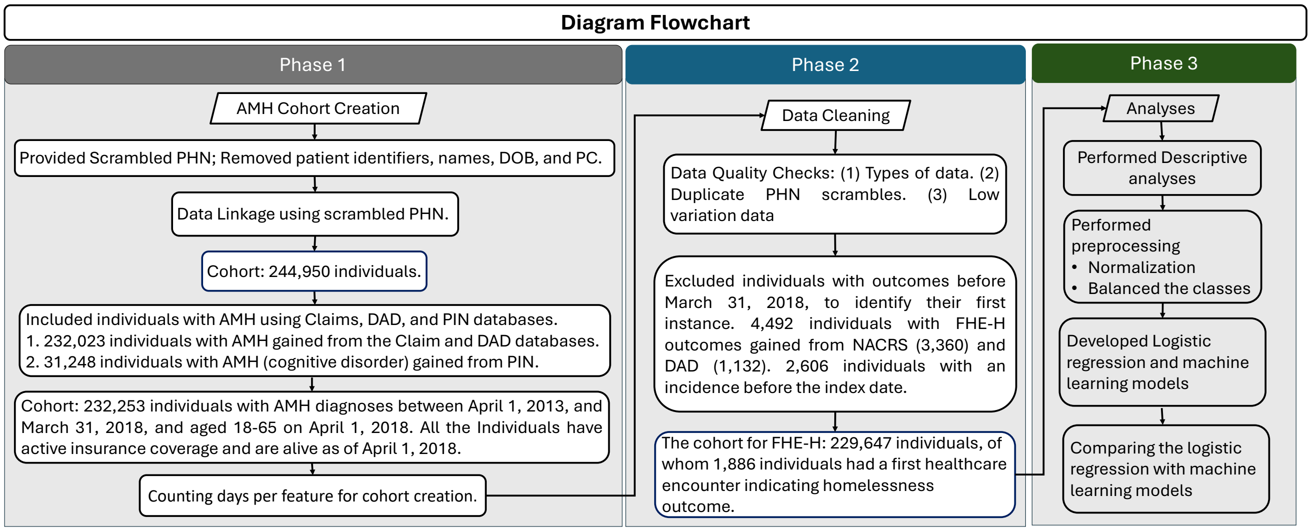


Abbreviations: AMH, addiction or mental health; PHN, personal health number; DOB, date of birth; PC, postal code; Claims, practitioner claims; DAD, discharge abstract database; PIN, pharmaceutical information network; FHE-H, first healthcare encounter indicating homelessness.
