# Supplementary figures and images for: Risk factors and predictive performance for first healthcare encounter indicating homelessness using administrative data among Calgary residents diagnosed with addiction or mental health conditions
Source: PLOS Digit Health. 2025 Oct 31;4(10):e0001064. doi: 10.1371/journal.pdig.0001064 (PMC12578244; doi:10.1371/journal.pdig.0001064)

**S4 Appendix:** The Framework for observation and prediction windows with a fixed index date.

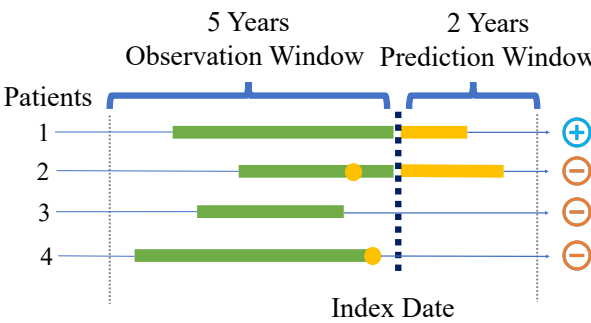

Supplement: S4 Appendix — (PDF) [file pdig.0001064.s004.pdf]
