# Supplementary material for: Risk factors and predictive performance for first healthcare encounter indicating homelessness using administrative data among Calgary residents diagnosed with addiction or mental health conditions
Source: PLOS Digit Health. 2025 Oct 31;4(10):e0001064. doi: 10.1371/journal.pdig.0001064 (PMC12578244; doi:10.1371/journal.pdig.0001064)
Supplement: S5 Appendix — (PDF) [file pdig.0001064.s005.pdf]

S5 Appendix: The outcomes and definitions

Data Source	ICD	Codes	Definition
DAD & NACRS	ICD-10-CA for "homelessness" and Inadequate housing"	Z590	Homelessness: to identify individuals who are without a place to live, capturing the situation of homelessness more precisely.
		Z591	Inadequate housing: to identify individuals living in housing that is insufficient or substandard, indicating inadequate living conditions
Claims	ICD-9-CM for "homelessness" and Inadequate housing"	V600	Lack of housing: to indicate that the individual does not have a fixed, regular, and adequate nighttime residence, essentially identifying a person as homeless.
		V601	Inadequate housing: to denote that the individual is living in housing that does not meet minimal standards of habitability, such as substandard or overcrowded conditions.
