# Supplementary material for: Risk factors and predictive performance for first healthcare encounter indicating homelessness using administrative data among Calgary residents diagnosed with addiction or mental health conditions
Source: PLOS Digit Health. 2025 Oct 31;4(10):e0001064. doi: 10.1371/journal.pdig.0001064 (PMC12578244; doi:10.1371/journal.pdig.0001064)
Supplement: S6 Appendix — (PDF) [file pdig.0001064.s006.pdf]

S6 Appendix: Predictors that were used in multivariable analysis.

Category	Predictors	Dichotomous values	Multinomial values
Sex	Male	B	B
	Female	Reference	Reference
Age category	18-29	B	B
	30-39	Reference	Reference
	40-49	B	B
	50-59	B	B
	60+	B	B
AMH conditions	Substance use disorder	B	CF
	Psychotic disorder	B	CF
	Mood disorder	B	CF
	Other psychiatric disorders	B	CF
	Anxiety disorder	B	CF
	Cognitive disorders	B	CF
	Deliberate self-harm	B	CF
Health service utilization	ED MH	B	CF
	ED non-MH	B	CF
	Non-MH hospitalized	B	CF
	General practitioner	B	CF
	ED (general)	B	CF
	Psychiatrist	B	CF
	MH hospitalized	B	CF
	Other physicians	B	CF
	Internal medicine	B	CF
	Neurologist	B	CF
Elixhauser comorbidities	Congestive Heart Failure	B	CF
	Cardiac Arrhythmias	B	CF
	Valvular Disease	B	CF
	Pulmonary Circulation Disorders	B	CF
	Peripheral Vascular Disease	B	CF
	Uncomplicated Hypertension	B	CF
	Complicated Hypertension	B	CF
	Paralysis	B	CF
	Other Neurological Disorders	B	CF
	Chronic Pulmonary Disease	B	CF
	Uncomplicated Diabetes	B	CF
	Complicated Diabetes	B	CF
	Hypothyroidism	B	CF
	Renal Failure	B	CF
	Liver Disease	B	CF
	Peptic Ulcer Disease (excluding bleeding)	B	CF
	HIV/AIDS	B	CF
	Lymphoma	B	CF
	Metastatic Cancer	B	CF
	Solid Tumor without Metastasis	B	CF
	Rheumatoid Arthritis / Collagen Vascular Diseases	B	CF
	Coagulopathy	B	CF
	Obesity	B	CF
	Weight Loss	B	CF
	Fluid and Electrolyte Disorders	B	CF
	Blood Loss Anemia	B	CF
	Deficiency Anemia	B	CF
	Alcohol Use	B	CF
	Substance misuse	B	CF
	Psychoses	B	CF
	Depression	B	CF
	Stroke	B	CF
	Dyslipidemia	B	CF
	Sleep Disorders	B	CF
	Ischemic Heart Disease	B	CF
	Seizure Disorders	B	CF
	Other Head Injury	B	CF
	History of Falls	B	CF
	Urinary Incontinence	B	CF
	Visual Impairment	B	CF
	Hearing Impairment	B	CF
	Tobacco Use	B	CF
	Delirium	B	CF
	Multiple Sclerosis	B	CF
	Parkinson's Disease	B	CF

Abbreviations: AMH, addiction or mental health; ED, emergency department; MH, mental health. Predictors were represented in the model using two formats: B (binary format, 0/1) to indicate the presence or absence of a condition, and CF (count format, $0 \rightarrow N$) to reflect the number of times a condition was recorded. For categorical variables, one category was used as the reference (e.g., "female" for sex), so if all other categories were coded as 0, it implied the reference category was present.
