# Supplementary material for: Risk factors and predictive performance for first healthcare encounter indicating homelessness using administrative data among Calgary residents diagnosed with addiction or mental health conditions
Source: PLOS Digit Health. 2025 Oct 31;4(10):e0001064. doi: 10.1371/journal.pdig.0001064 (PMC12578244; doi:10.1371/journal.pdig.0001064)
Supplement: S7 Appendix — (PDF) [file pdig.0001064.s007.pdf]

S7 Appendix: The hyperparameters for each machine learning model.

Model	optimum hyperparameters	Experimented hyperparameters	Number of examinations
RF	bootstrap= 'TRUE'<br>max_depth= 10<br>max_features= 'log2<br>min_samples_leaf= 2<br>min_samples_split= 2<br>n_estimators= 1500<br>criterion= 'gini'	bootstrap=('True')<br>max_depth=(10 20 30 40 50 60 70 80 90 100)<br>max_features=('sqrt' 'log2' 'None')<br>min_samples_leaf=(1 2 4 8)<br>min_samples_split=(2 5 10)<br>n_estimators=(500 800 1000 1500 2500 5000)<br>criterion=('gini' 'entropy' 'log_loss')	4,320
XGBoost	max_depth=6<br>learning_rate=0.001<br>subsample=0.9<br>colsample_bytree=0.7<br>colsample_bylevel=0.9<br>n_estimators=2500	max_depth= (3 5 6 10 15 20)<br>learning_rate=(0.0001 0.001 0.01 0.1)<br>subsample=(0.5 0.7 0.8 0.9 1.0)<br>colsample_bytree=(0.4 0.5 0.6 0.7 0.8 0.9 1.0)<br>colsample_bylevel=(0.4 0.5 0.6 0.7 0.8 0.9 1.0)<br>n_estimators=(100 500 800 1000 1500 2500 5000)	9,720

Abbreviations: RF, Random Forest; XGBoost, Extreme Gradient Boosting.
