# Supplementary material for: Risk factors and predictive performance for first healthcare encounter indicating homelessness using administrative data among Calgary residents diagnosed with addiction or mental health conditions
Source: PLOS Digit Health. 2025 Oct 31;4(10):e0001064. doi: 10.1371/journal.pdig.0001064 (PMC12578244; doi:10.1371/journal.pdig.0001064)
Supplement: S8 Appendix — (PDF) [file pdig.0001064.s008.pdf]

S8 Appendix: Summary of Individuals with AMH and Cohort Exclusions.

DAD & Claim (NI with AMH)	PIN (NI with AMH)	Total NI with AMH	removed NI (FHE-H before ID)	**Cohort**
232,023	31,248	232,253	2,606	***229,647***

Abbreviations: AMH, addiction or mental health; Claims, practitioner claims; DAD, discharge abstract database; PIN, pharmaceutical information network; FHE-H, first healthcare encounter indicating homelessness; NI, number of individuals; ID, index date.
